# Applying Trauma‐Informed Principles to Support Learner Engagement and Wellbeing in UK Undergraduate Medical Education: A Qualitative Study

**DOI:** 10.1111/tct.70540

**Published:** 2026-10-27

**Authors:** Helen West, Dominic Johnson

**Affiliations:** ^1^ Department of Psychology University of Liverpool Liverpool UK; ^2^ UCL Medical School, Faculty of Medical Sciences University College London London England

**Keywords:** medical education, qualitative, trauma, trauma‐informed, undergraduate

## Abstract

**Introduction:**

Trauma‐informed education recognises the impact of psychological trauma and seeks to create environments that promote wellbeing and minimise further harm. In medical education, this approach has the potential to enhance student engagement, learning, professional development and overall health. This study explored how trauma‐informed principles (safety, trust, support, collaboration, empowerment and social justice) are currently applied in undergraduate medical education and identified opportunities for broader implementation.

**Methods:**

Thirty‐eight medical educators from six UK medical schools participated in interactive workshops. Participants selected trauma‐informed principles to discuss in small groups, guided by the prompts ‘What are we already doing?’ and ‘What else could we do?’ Data were analysed using template analysis.

**Results:**

Six themes were identified that guide the application of a trauma‐informed approach in undergraduate medical education: recognising student diversity and individuality; fostering connection and belonging; managing sensitive or challenging content; considering clinical experiences; involving students in decision‐making; and the role of educators.

**Discussion:**

Most participants were unfamiliar with trauma‐informed approaches in medical education and valued the opportunity to reflect and share practices. Given that many medical students have experienced trauma or may do so during their training, integrating trauma‐informed principles can help to mitigate its impact and support students.

**Conclusion:**

Trauma‐informed principles promote equity, engagement and wellbeing, offering a valuable lens for improving medical education.

## Practitioner Points

- Many medical students have experienced trauma or will do so during their studies, which can disrupt emotional regulation and hinder learning.
- Trauma‐informed education promotes equity, engagement and wellbeing, equipping students to become compassionate clinicians and deliver better patient care.
- This study reinforces the value of trauma‐informed principles for improving medical education, outlines practical strategies for implementation and emphasises the importance of professional development for educators in this area.

## 1 Introduction

Psychological trauma can have enduring impact on an individual's wellbeing and functioning. It is defined as ‘an event, series of events, or set of circumstances that is experienced by an individual as physically or emotionally harmful or life threatening and that has lasting adverse effects’ [1]. These experiences are prevalent among student populations, and studies suggest that up to 85% of individuals have encountered a traumatic event by university age [2]. Published evidence on the rate of trauma among medical students is limited and varies in quality [3]. US studies show high rates of adverse childhood experiences (ACEs), with 51%–74% of medical students reporting at least one ACE and 12%–25% reporting four or more [4, 5]. Measures such as ACEs capture the occurrence of specific adverse events, not an individual's subjective experience or the personal impact of those events. As a result, they are imperfect indicators of trauma; however, they do highlight that trauma is widespread. These adversities disproportionately affect students from socially disadvantaged and minoritised backgrounds, compounding existing structural inequalities [6].

Medical students experience higher levels of stress, anxiety and psychological distress than others their age [7], affecting academic performance, professional development, empathy and career trajectories [8]. Medical education frequently exposes students to emotionally sensitive material and clinical situations that may evoke distress, trigger previous experiences or lead to vicarious traumatisation [9]. While trauma depends on an individual's unique experience of an event, some content is more likely to activate trauma responses for learners, such as topics related to violence, abuse or sensitive diagnoses [10].

Trauma has been linked to psychological distress in health professions students [6] and is associated with burnout in medical professionals [11]. Traumatic experiences can impair students' mental and physical health, relationships, academic performance and progression [12]. Emerging evidence that trauma may influence medical students' career motivations and specialty choice suggests effects that extend beyond personal wellbeing into professional identity formation [5]. Trauma produces neurobiological changes in brain regions responsible for attention, memory and emotional regulation [13]. When students experience emotional dysregulation, cognitive resources required for concentration, information retention and reasoning are reduced, restricting learning effectiveness [14, 15].

A trauma‐informed approach to learning has gained increasing attention in higher education [14] and medical education [16] as a framework for supporting learner wellbeing and education. Trauma‐informed practice acknowledges the prevalence of trauma, recognises signs and symptoms, responds in practice and policy and seeks to prevent re‐traumatisation [1]. The six core principles of this approach (see Figure 1) apply across curriculum design, teaching methods, assessment, institutional culture, student support and the broader learning environment [16]. Importantly, trauma‐informed education moves beyond focusing on individual resilience to address structural and cultural contributors to student distress.

**FIGURE 1:**
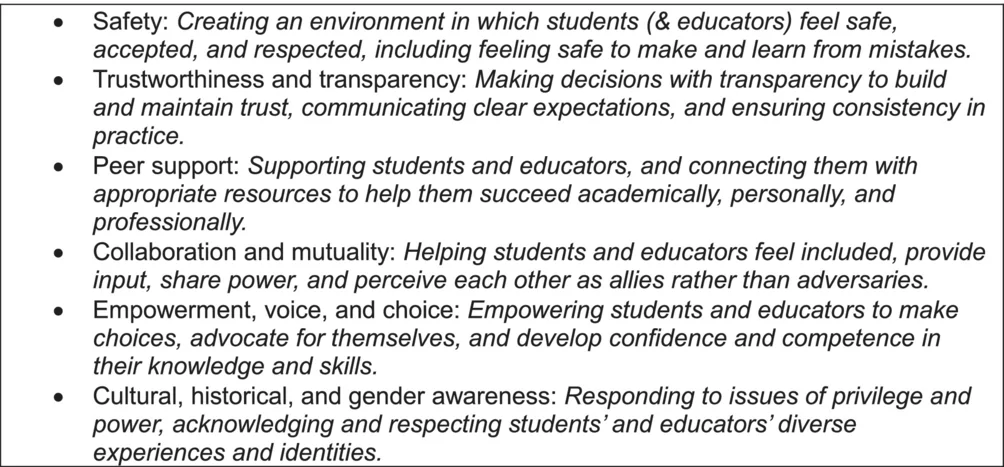
The six core principles of a trauma‐informed approach [1] with prompts in italics based on the Trauma‐Informed Teaching Toolbox [17].

Applying trauma‐informed principles to medical education has the potential to enhance engagement, wellbeing, learning and professional development by fostering psychological safety, empowerment and inclusive learning environments [18]. While this study focuses on the educational context, i.e., how teaching practices, interactions and environments can align with trauma‐informed principles, it is also essential that medical students gain competency in applying trauma‐informed care in their own clinical practice [16].

*Applying trauma‐informed principles to medical education has the potential to enhance engagement, wellbeing, learning and professional development by fostering psychological safety, empowerment and inclusive learning environments*.

The principles of a trauma‐informed approach offer a comprehensive framework for supporting medical students who have experienced trauma, reducing exposure to potentially traumatic experiences during training and preventing re‐traumatisation [16]. This study explores the research questions: How are trauma‐informed principles currently applied in undergraduate medical education from the perspective of educators? and What opportunities exist to expand their use?

*The principles of a trauma‐informed approach offer a comprehensive framework for supporting medical students who have experienced trauma, reducing exposure to potentially traumatic experiences during training and preventing re‐traumatisation*.

## 2 Methods

### 2.1 Methodology

This research study is based on a critical realist paradigm, recognising that our experience of reality is socially located [19]. Participatory workshops were used to provide a broad overview of current practice. Participant responses were understood to represent a shared understanding of that reality, acknowledging the social constructivist position that subjective meanings are formed through social norms and interactions with others, including while participating in data collection workshops.

We chose to use template analysis, a form of thematic analysis, because this technique provides a structured process for analysing textual data reflecting a socially contextualised reality [20]. Template analysis can both be informed by prior theory and generate unanticipated insights, so it was suitable for topic where there is an existing theoretical framework but limited empirical research.

The research was conducted by a psychology lecturer, and a senior lecturer who is an educator and psychiatrist. As White British people, with our own experiences of education, educating and trauma, we were aware that we viewed the data through specific lenses and reflected on how this influenced our interpretation and analyses.

### 2.2 Ethical Approval

Approval was granted by the University of Liverpool Research Ethics Committee (Reference: 12813).

### 2.3 Participants and Recruitment

Individuals working in medical education were invited to participate in four workshops held between November 2023 and May 2024. One workshop was part of a regional conference programme, and delegates were provided with information about the study in advance. Three workshops were held within a medical school, and invitations to participate were distributed via staff briefings.

### 2.4 Procedure

Potential participants were informed about the study and invited to participate. Workshops included a brief introduction including defining trauma, the prevalence and effects among students and the principles of a trauma‐informed approach using the prompts shown in Figure 1. Following Edelman's [21] guidance to assume that anyone may have experienced trauma, we did not ask participants about personal trauma histories. Workshops were intentionally designed using a trauma‐informed approach [22], demonstrating the principles in action to ‘model the model’ [21]. This approach fostered a safe and inclusive environment, allowing participants to engage meaningfully without disclosing personal experiences.

Participants chose which principles to discuss in groups of 3–5 participants. They were asked ‘What are you already doing?’ and ‘What else could you do?’ and added written comments. These small group discussions were followed by brief feedback to the whole group. This process was repeated, so that groups selected and discussed at least two principles. Participants completed a brief feedback survey at the end of the workshop. All comments were transcribed and sent to participants after the workshop to check and submit any additional comments. Data was stored securely, in line with the University of Liverpool Research Data Management Policy.

### 2.5 Analysis

The six steps of template analysis [20] were used to analyse the dataset (Table 1). We used strategies to establish trustworthiness [23] (Table 2) and SRQR reporting standards [24] (Supporting Information).

**TABLE 1:** The process of template analysis in this study.

1. Familiarisation with the dataset	The authors read through the dataset in full and made notes during this process of familiarisation.
2. Preliminary coding of the data	We highlighted data from Workshop 1 that contributed to our understanding. We considered using the principles of a trauma‐informed approach as a priori themes, however we had noted that related comments were made with reference to different principles. We therefore decided to explore these cross‐cutting themes, rather than the principles, which had been discussion prompts for participants.
3. Organising codes into initial themes	Clusters of codes in Workshop 1 were organised into themes, particularly noting similar comments that were made when discussing different principles.
4. Defining an initial coding template	We developed an initial version of the coding template based on the dataset from Workshop 1. This workshop included educators with a wide variety of roles, years of experience and medical schools, so we felt that it captured a good cross‐section of the comments covered in the whole dataset.
5. Applying and modifying the template	The initial template was applied to Workshops 2, 3 and 4, coding data to existing themes where relevant. Some of the new data did not readily ‘fit’ the template, therefore a new theme was added (Clinical).
6. Finalising the template	The modified template was applied to all data, completing this iterative process. Subthemes were identified within the template themes, and lateral relationships that permeate distinct clusters were noted as ‘integrative themes’.

**TABLE 2:** Strategies used to establish rigour and trustworthiness in this study [23].

Credibility	The credibility of analysis was strengthened by involving two researchers in developing, reviewing and defining themes, and producing the report. Their different perspectives provided triangulation and increases the trustworthiness of the findings.
Transferability	We recognise that medical education practices vary and that participants mainly reflected on their local context. Including participants from diverse roles and medical schools increases the transferability of findings. We have described the study's context to help readers evaluate whether the findings are relevant to other settings.
Dependability	We have thoroughly described the data collection process and steps involved in template analysis. We documented meeting notes and key decisions made during analysis, to provide a transparent audit trail of the process. The data is available from the University of Liverpool Research Data Catalogue.
Confirmability	The researchers discussed their interpretations of the data, with opportunities for reflection. The rationale behind theoretical, methodological and analytical choices have been described following the SRQR reporting standards [24] (Supporting Information) to ensure transparent reporting.

## 3 Results

A total of 38 individuals working in medical education participated in workshops (Table 3). Three workshops comprised staff at the University of Liverpool, one was at a regional conference. Three workshops were in person (*n* = 12, 9, 12), one was online (*n* = 5). A total of 24 participants were female; 14 were male. Most participants (*n* = 30) were staff at the University of Liverpool or related Trusts, however participants from five other Universities took part in the regional workshops (*n* = 8). Participants had worked in medical education for under 1 year to 24 years (median = 6 years, mean = 8.4 years). Their roles included academic and clinical positions, from early career roles to senior leadership.

**TABLE 3:** Characteristics of workshop participants.

	Workshop 1	Workshop 2	Workshop 3	Workshop 4	Total
Workshop setting	In person, regional conference	Online	In person, University campus	In person, University campus	
Number of participants	12	5	9	12	38
Number of medical schools represented	6	1	1	1	6
Gender					
Male	4	1	3	6	14
Female	8	4	6	6	24
Years in medical education					
Median (range)	6.5 (0.25–17)	15 (4–20)	2 (0.1–10)	8 (1–24)	6 (0.1–24)
Aware of trauma‐informed education					
Yes	3	1	0	2	6
In another context	1	3	2	0	6
No	8	1	7	10	26

Six integrative themes and 16 subthemes were identified from the analysis (Figure 2).

**FIGURE 2:**
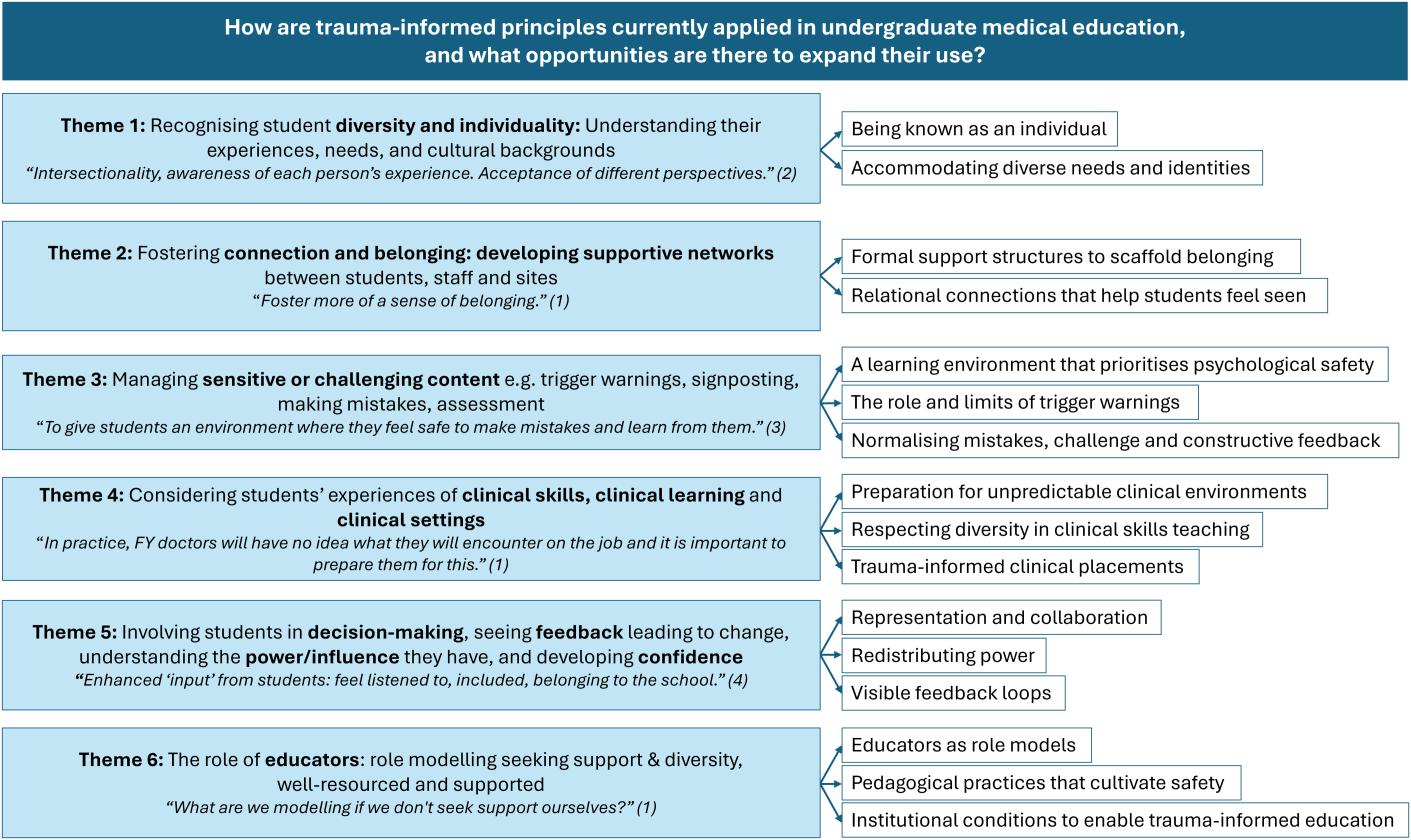
Themes and subthemes identified from template analysis of workshop data.

### 3.1 Theme 1: Recognising Student Diversity and Individuality: Understanding Their Experiences, Needs and Cultural Backgrounds

#### 3.1.1 Being Known as an Individual

Educators emphasised that knowing students as individuals enables them to identify and respond to trauma: ‘Getting to know students‐asking about things outside of medicine‐extracurricular, plans for weekend’ (workshop 3). Learning students' names and asking about their interests helped build trust, belonging and psychological safety, enabling earlier identification of distress. While large cohorts make this challenging, small group teaching and academic advisor systems facilitated continuity and connection: ‘Large cohort: only know those who stand out (for good and bad reasons)‐lack of connection’ (workshop 1).

#### 3.1.2 Accommodating Diverse Needs and Identities

Participants described adapting teaching to accommodate students' diverse needs and identities, including differences in prior knowledge and experience, personality (e.g., quieter students), cultural and religious background, caregiving roles, disabilities and experiences of trauma and discrimination: ‘Be more inclusive with students' varying backgrounds and identities’ (workshop 4). They highlighted that students from minoritised groups are more likely to experience trauma, acknowledging the intersectionality of identities. Educators described signalling inclusivity through explicit discussion and embedded practice, to build the safety and trust required for engaging with sensitive material, ‘Encouraging mutual respect and understanding’ (workshop 1). ‘Students voicing their diverse experiences/identities so other students acknowledge them’ (workshop 1).

Overall, this theme communicates that when students experience relational safety and support for their diverse identities and needs, they are more able to engage with emotionally demanding content.

### 3.2 Theme 2: Fostering Connection and Belonging: Developing Supportive Networks Between Students, Staff and Sites

#### 3.2.1 Formal Support Structures to Scaffold Belonging

Participants identified formal systems (e.g., tutor schemes, wellbeing services, workshops on wellbeing topics, transfer of support information to supervisors, structured transitions) as fostering belonging, recognising trauma and providing support. These systems proactively create a network of support for the whole cohort, while also providing safety for individuals who need additional intervention due to past trauma or who are reluctant to seek support. ‘Making advice available to those who don't reach out for support in person’ (workshop 1). Participants described that effective support relies on clear communication, signposting and coordination (e.g., linking tutors with wellbeing services, providing financial advice, ensuring accessible referral processes).

#### 3.2.2 Relational Connections That Help Students Feel Seen

In addition to formal support systems, informal staff‐student connections were viewed as central to trauma‐informed education. Strategies to help students feel seen and supported included social activities, peer support and mentoring, reverse mentoring and collaboration: ‘Staff‐student social spaces and collaborative areas’ (workshop 4). Induction was seen as critical in establishing this culture, while consistent small group teaching supported ongoing relational connection, and was viewed as protective for students with trauma histories: ‘Small size group, same group all year, same educator, clinician who is doing the job, safety in authenticity’ (workshop 2).

The overarching concept of this theme is that trauma‐informed education requires environments where students feel connected, known and supported through both formal systems and everyday relational practices.

### 3.3 Theme 3: Managing Sensitive or Challenging Content, e.g., Trigger Warnings, Signposting, Making Mistakes, Assessment

#### 3.3.1 A Learning Environment That Prioritises Psychological Safety

Participants discussed using trauma‐informed strategies when teaching sensitive content (e.g., mental health problems, racism, palliative care, emotionally charged simulations). Approaches included advance notice, opportunities for one‐to‐one discussions and permission to leave the room if needed: ‘Ability to leave and rejoin sessions when suitable’ (workshop 4). Psychological safety was supported by continuity with tutors and peers, playful or reflective teaching approaches, and opportunities for debrief. However, participants cautioned that debriefing alone may be insufficient: ‘Just because you've done a debrief doesn't mean you've dealt with it’ (workshop 2), as superficial containment may mask ongoing distress.

#### 3.3.2 The Role and Limits of Trigger Warnings

Trigger warnings were viewed as helpful for preparing students and reducing re‐traumatisation without requiring disclosure of trauma histories. ‘Trigger warnings. Builds trust‐students know what to expect for example in a teaching session, images they might see that could be distressing’ (workshop 1). However, participants acknowledged limitations, including potentially increasing anxiety, unpredictability of distress and shifting responsibility onto students: ‘Say they're fine but something bubbles up even when prepared and warned’ (workshop 2). Participants emphasised the need to support students in recognising and managing distress through adaptive coping strategies and emotional regulation rather than avoidance.

#### 3.3.3 Normalising Mistakes, Challenge and Constructive Feedback

Participants viewed creating a safe space for making mistakes and being challenged as essential to reduce distressing experiences and minimise re‐traumatisation: ‘Allow students to make mistakes‐trauma‐informed prescribing over anxiety about risks of prescribing’ (workshop 1). Participants advocated for formative assessments, tutor transparency to normalise failure as part of learning, and modelling feedback as a constructive process.

This theme illustrates that trauma‐informed teaching of sensitive content extends beyond warnings and debriefs to include relational safety, transparent communication and pedagogical approaches that balance safety and challenge.

*Trauma‐informed teaching of sensitive content extends beyond warnings and debriefs to include relational safety, transparent communication and pedagogical approaches that balance safety and challenge*.

### 3.4 Theme 4: Considering Students' Experiences of Clinical Skills, Clinical Learning and Clinical Settings

#### 3.4.1 Preparation for Unpredictable Clinical Environments

Simulation was seen as a supportive setting in which students can practise and make mistakes, reducing future distress in clinical settings. However, participants noted its limits in replicating real clinical environments: ‘You cannot “sanitise” all educational provision as this is not representative of working in the health setting in real life’ (workshop 2). The limitations of trigger warnings were considered particularly salient to the unpredictability of clinical learning. Suggested strategies included reducing the use of trigger warnings as students progress and equipping students with self‐regulation skills: ‘Helping students to “sit with” their triggers and ways to support students with this in a way that doesn't necessarily mean they have to leave the educational or clinical environment’ (workshop 2).

#### 3.4.2 Respecting Diversity in Clinical Skills Teaching

Inclusive practice in clinical skills teaching was viewed as trauma‐informed, recognising that students with minoritised identities or trauma histories may find aspects of learning in this environment particularly challenging. Examples included using privacy booths, choice of partners for peer physical examinations, and ensuring scenarios and equipment reflect real‐world diversity: ‘We have scenarios and equipment that tries to be diverse’ (workshop 4).

#### 3.4.3 Trauma‐Informed Clinical Placements

Clinical placements may expose students to distressing or retraumatising experiences. Participants emphasised the need for safe spaces for reflection and discussion. ‘Clinical debrief sessions: same tutor for 12 weeks. Students can set topic each week based on experiences on wards’ (workshop 1). Trauma‐informed placements depend on inclusive decision‐making and effective transfer of information to support students who have experienced trauma. This might include tailoring placements to minimise re‐traumatisation, for example when a student's personal experience is closely related to the specialty: ‘Palliative care, difficult conversations, suicidal patient. Offering students the option to step out of the room’ (workshop 2). Suggestions included involving students in communication with Trust sites, supporting student advocacy and engaging hospital clinicians in educator development.

This theme highlights how clinical environments require scaffolding to help students navigate unpredictable and emotionally demanding experiences while developing competence and confidence.

### 3.5 Theme 5: Involving Students in Decision‐Making, Seeing Feedback Leading to Change, Understanding the Power/Influence They Have and Developing Confidence

#### 3.5.1 Representation and Collaboration

Student representation on medical school committees was seen as key to collaboration and visibility. Participants suggested holding working groups to address issues collectively and empower students (‘Working groups – open and honest discussions with students about what we teach and how’ (workshop 4)), while noting that dominant voices may not represent all students. ‘Does the loud person speak for the larger population?’ (workshop 1).

#### 3.5.2 Redistributing Power

As trauma involves a loss of control, enabling student influence learning was seen as restorative. Educators acknowledged inherent and perceived power dynamics and sought to create more equal relationships. ‘Giving students the choice of how they want their session to run in order to develop confidence in their own knowledge and skills’ (workshop 1). Building confidence was also seen as a way to empower students and prevent trauma.

#### 3.5.3 Visible Feedback Loops

Participants highlighted the importance of demonstrating change in response to feedback to build trust and transparency. ‘Enhanced “input” from students: feel listened to, included, belonging to the school’ (workshop 4). However, feedback processes could also be harmful or difficult to action if poorly managed. ‘Mutual respect: Lectures on how to give feedback. How that effects the person receiving feedback’ (workshop 1).

Overall, this theme considers how trauma‐informed education requires student agency, visible responsiveness and attention to power dynamics to foster safety, confidence and belonging.

### 3.6 Theme 6: The Role of Educators: Role Modelling Seeking Support and Diversity, Well‐Resourced and Supported

#### 3.6.1 Educators as Role Models

Participants emphasised that educator wellbeing, authenticity and emotional regulation underpin a trauma‐informed approach. Educators model behaviours such as help‐seeking, reflection and learning from mistakes, which foster trust and connection. ‘Widening wellbeing focus to staff as well as students’ (workshop 1). ‘Awareness that we all have biases, therefore modelling in interaction that becoming aware of them is an aspect of growth, rather than the narrative of being accused’ (workshop 2). Diverse educators were seen as especially impactful role models. ‘How can you be what you can't see? How do you showcase wide variety of educator backgrounds?’ (workshop 1).

#### 3.6.2 Pedagogical Practices That Cultivate Safety

Effective teaching techniques were seen as central to psychological safety, including managing group dynamics, encouraging participation and allowing space for mistakes and disagreement. High‐quality teaching also supports student confidence in their knowledge and was viewed as protective against future trauma. ‘Educational quality is part of safety, the broad approach can be damaging. Long term impact: shaky knowledge & imposter syndrome’ (workshop 2).

#### 3.6.3 Institutional Conditions to Enable Trauma‐Informed Education

Participants commented that implementing trauma‐informed approaches depended upon institutional support, including training, peer observation, sharing resources and stronger links with clinical settings. Key training areas included microaggressions, support pathways and inclusive culture. Participants recognised that many trauma‐informed practices were already in use but benefited from clearer framing. ‘I have used the approach within my teaching without realising that it was a formal definition’ (workshop 4).

Overall, this theme emphasises that trauma‐informed education depends on educator relationships, emotions and behaviours, and institutional conditions that enable safe, supportive learning environments.

## 4 Discussion

These findings demonstrate that many trauma‐informed practices are already in use, often without recognising that this is the case, and highlight further ways in which they can be applied. The trauma‐informed framework helped participants re‐evaluate existing practices and identify areas for development, emphasising strategies to recognise and respond to trauma in ways that reduce harm and re‐traumatisation.

The themes identified were: Understanding the individuality and diversity of students; Fostering connections, belonging and supportive networks; Considering how to handle sensitive or challenging content; Considering clinical experiences; Student involvement in decision‐making; and the role of educators. These themes offer strategies to operationalise trauma‐informed principles, which can initially appear abstract, in the context of healthcare education. Findings identify practical ways to better support medical students who have experienced trauma, which include providing consistent small‐group learning, strong formal support systems and personalised placement experiences. It outlines how to reduce students' exposure to potentially traumatic situations, such as by ensuring psychological safety, offering effective clinical debriefing, preparing students for challenging environments and training staff in trauma‐informed practices. Finally, the results highlight approaches to prevent re‐traumatisation, including accommodating diverse learning needs, using trigger warnings, and promoting shared decision‐making between educators and students. This study builds on prior work which proposed strategies such as preparation, trigger warnings, titration of sensitive material, student autonomy and self‐care [16, 25, 26, 27].

With increasing diversity among medical students and increasing cohort numbers [28], a greater number of students will potentially have experienced trauma. It is therefore imperative that diverse needs are accommodated, mechanisms exist for students to be known individually, and that formal and informal structures are resourced to support students. This is especially important for students from minoritised groups, who may have greater exposure to trauma and potential for re‐traumatisation [6].

Most interventions to support medical student mental health focus on individual wellbeing, such as formal support services or mindfulness training [29]. In contrast, trauma‐informed approaches emphasise cultural and organisational change by promoting safety, trust, collaboration and shared power [18, 21]. This includes involving students in decision‐making, using transparent and respectful communication and assessment processes, and maintaining continuity with trusted tutors and peers. These upstream strategies are crucial for supporting students who have experienced trauma, reducing the likelihood of traumatic events and minimising re‐traumatisation.

*Trauma‐informed approaches emphasise cultural and organisational change by promoting safety, trust, collaboration and shared power*.

As students progress from classroom to clinical settings, they must learn to manage emotional responses to distressing experiences. A trauma‐informed approach can scaffold the development of emotional regulation. Research supports the role of adaptive emotional regulation in protecting healthcare professionals' mental health and enhancing patient care [30, 31]. However further research is needed on effective teaching strategies for emotional regulation.

### 4.1 Strengths and Limitations

This UK‐based study provides novel insights into trauma‐informed medical education, generating a rich dataset that distils many areas of good practice. Most participants were unfamiliar with trauma‐informed education, and feedback indicated that they found the workshops valuable, underscoring the need for professional development [32]. A trauma‐informed approach is inherently contextual and relational; therefore, while certain strategies are typically helpful, educators must adapt principles to their specific environment for meaningful implementation [18]. Social dynamics inevitably shaped workshop discussions; however we view collective meaning‐making and the construction of a shared understanding of reality as integral to this methodology, rather than a hindrance. Likewise, researcher subjectivity is embraced as a contextual resource for knowledge generation, rather than a limitation to be managed.

Several strategies were employed to establish rigour and trustworthiness [23] (Table 2), including data analysis and theme development by authors from different disciplines. While tools like post‐it notes and Padlet boards supported accessibility, verbatim transcription could have generated more nuanced data. Interview or focus group studies could deepen understanding and develop additional trauma‐informed strategies. Limitations include the predominance of University‐based clinical educators. Although participants represented six medical schools, the majority were from a single institution. It was beyond the scope of this study to consider curricular content relating to trauma impacts on patients and skills for trauma‐informed care delivery, and this has been considered elsewhere [33]. This study provides a foundation for research across disciplines, including nursing, allied health and dentistry, involving students and placement clinicians to capture broader perspectives.

## 5 Conclusion

Many medical students have experienced trauma or will do so during their studies, which can disrupt emotional regulation and learning. This study confirms that trauma‐informed principles offer a valuable lens for improving medical education and highlights educators' need for professional development on this topic. Through participatory workshops, we developed themes to apply these principles in practice, supporting student wellbeing, learning and professional development. Trauma‐informed education promotes equity, engagement and wellbeing; equipping students to become compassionate clinicians and deliver better patient care.

## Author Contributions

**Helen West:** conceptualisation, methodology, data curation, project administration, formal analysis, writing – original draft, writing – review and editing, visualisation. **Dominic Johnson:** conceptualisation, methodology, formal analysis, writing – original draft, writing – review and editing.

## Funding

The authors have nothing to report.

## Conflicts of Interest

The authors declare no conflicts of interest.

## Supporting information


**Data S1:** Supporting information.

## Data Availability

The data that support the findings of this study are openly available in University of Liverpool Research Data Catalogue at https://datacat.liverpool.ac.uk.
